# Exercise reverses learning deficits induced by hippocampal injury by promoting neurogenesis

**DOI:** 10.1038/s41598-020-76176-1

**Published:** 2020-11-06

**Authors:** Lavinia N. Codd, Daniel G. Blackmore, Jana Vukovic, Perry F. Bartlett

**Affiliations:** 1 Queensland Brain Institute, The University of Queensland, Brisbane, QLD 4072 Australia; 2 School of Biomedical Sciences, The University of Queensland, Brisbane, QLD 4072 Australia

**Keywords:** Adult neurogenesis, Neuroscience, Neurogenesis, Regeneration and repair in the nervous system

## Abstract

Hippocampal atrophy and cognitive decline are common sequelae of many neurodegenerative disorders, including stroke. To determine whether cognitive decline can be ameliorated by exercise-induced neurogenesis, C57BL/6 mice in which a unilateral hippocampal injury had been induced by injecting the vasoconstrictor endothelin-1 into their right hippocampus, were run voluntarily for 21 days on a running-wheel. We found the severe deficits in spatial learning, as detected by active place-avoidance task, following injury were almost completely restored in animals that ran whereas those that did not run showed no improvement. We show the increase in neurogenesis found in both the injured and contralateral hippocampi following running was responsible for the restoration of learning since bilateral ablation of newborn doublecortin (DCX)-positive neurons abrogated the cognitive improvement, whereas unilateral ablations of DCX-positive neurons did not prevent recovery, demonstrating that elevated neurogenesis in either the damaged or intact hippocampus is sufficient to reverse hippocampal injury-induced deficits.

## Introduction

Cognitive deficits are associated with neurodegenerative diseases and brain injuries, including stroke. Stroke survivors perform badly in acute tests of learning, memory, and cognition, and display accelerated long-term cognitive decline compared with non-stroke subjects^1^. Atrophy of the hippocampus, a major neurogenic region of the brain that plays a critical role in learning and memory, is common in humans following cortical or subcortical stroke and is frequently associated with impaired cognition^2–4^. The hippocampus is one of the few brain regions where the production of new neurons (neurogenesis) continues into adulthood in mammals, including in humans. We and others have shown that this neurogenesis is essential for contextual spatial learning and memory in mice^5–13^, and strategies to stimulate ongoing adult neurogenesis could potentially improve recovery.

The key regulator of neurogenesis in the hippocampus is the number of hippocampal precursors; these cells generally remain quiescent but distinct populations can be activated by different factors^14–16^, including exercise^11,17–20^, which has been associated with improved spatial learning^11,17^. Furthermore, several studies have reported that exercise results in improved cognitive performance in humans following stroke^21^. The short survival period and motor deficits that result from the recommended and commonly used middle cerebral artery occlusion (MCAO) rodent stroke model^22,23^ confound the accurate, long-term assessment of strategies to enhance recovery of hippocampal-dependent learning following ischemic injury. To address this issue, we developed a mouse model of unilateral hippocampal injury that results in a measurable learning deficit, but with prolonged survival and no motor impairment. Here we report that voluntary exercise after unilateral hippocampal injury leads to increased bilateral hippocampal neurogenesis and recovery in learning deficits, and that unilateral increased neurogenesis is sufficient for this recovery, regardless of whether this occurs in the injured or uninjured hippocampus.

## Results

### Intrahippocampal injection of ET-1 induces a localized lesion and learning deficit

**Figure 1:**
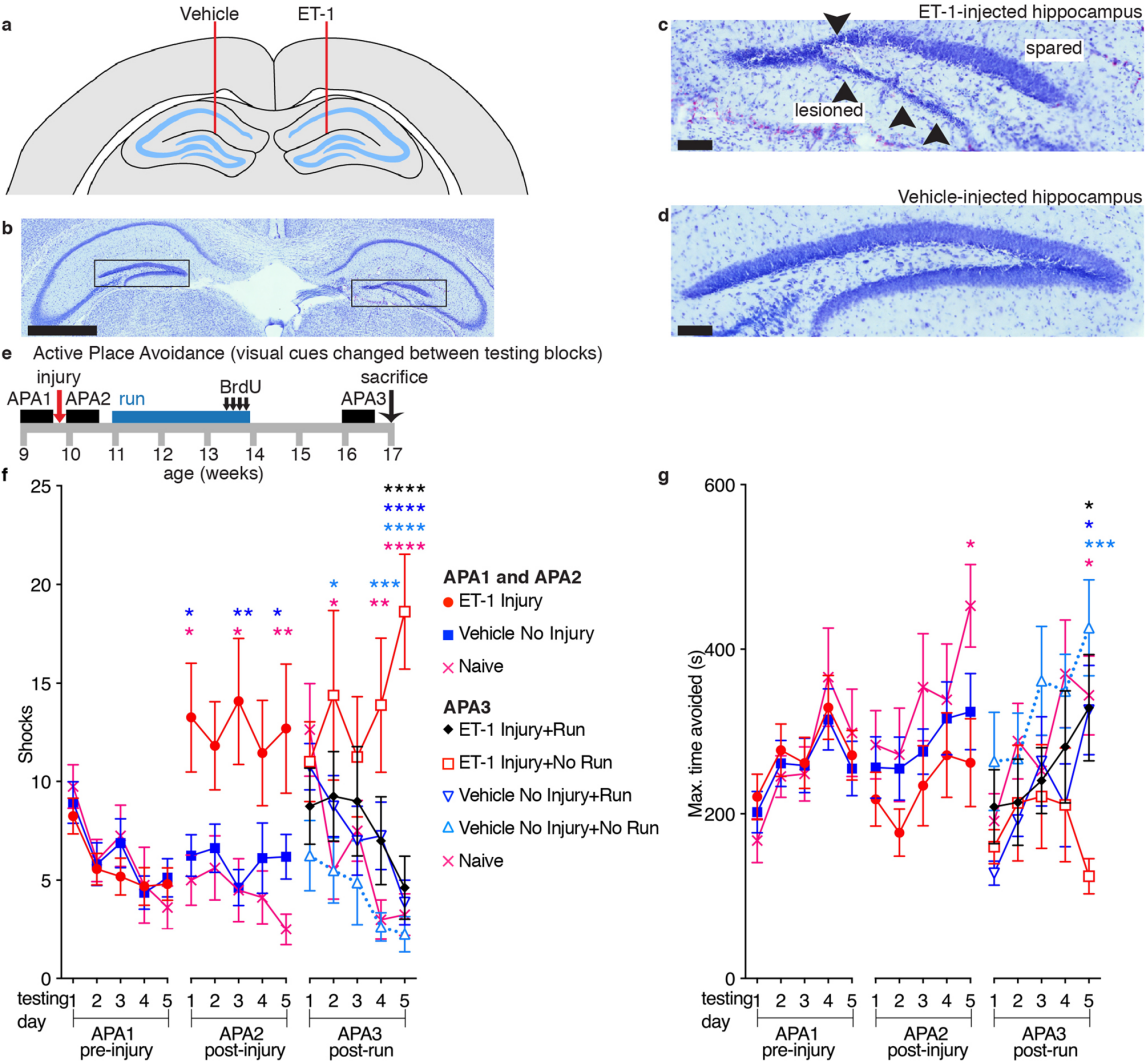
Unilateral intrahippocampal injection of ET-1 causes a hippocampal lesion and a learning deficit that is rescued by running. (**a**) Endothelin-1 (ET-1) was injected into the right hippocampus to induce stroke-like conditions (schematic adapted from Paxinos and Franklin, 2001^45^). Contralateral injection of PBS served as a control. Vehicle No Injury groups received bilateral injections of PBS. (**b**–**d**) Cresyl violet staining showing an ET-1-induced unilateral lesion (arrowheads indicate lesion; scale bars: (**b)**, 1 mm, (**c)** and** (d)**, 100 µm). (**e**) Experimental design. (**f** and** g**) Following unilateral ET-1 hippocampal injury, animals were unable to learn the active place avoidance (APA) shock-zone location but this learning deficit was rescued following voluntary running. ET-1 Injury animals that ran learned similarly to vehicle-injected No Injury or naïve animals and received significantly fewer shocks than ET-1 Injury No Run animals and the maximum time they avoided the shock-zone was significantly longer than ET-1 Injury No Run animals (mean ± SEM, *n* = 8/group; repeated measures two-way ANOVA and Bonferroni post hoc comparisons: APA 1–2 vs. ET-1 Injury; APA3 vs. ET-1 Injury + No Run; *p < 0.05; **p < 0.01; ***p < 0.001; ****p < 0.0001; colors show the comparison group).

**Figure 2:**
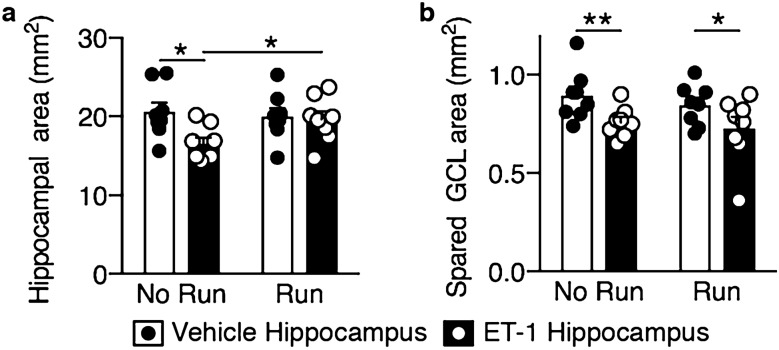
Unilateral ET-1 hippocampal injury reduced the hippocampal area and the spared area of the granule cell layer (GCL), but hippocampal area was increased with running. (**a**) The hippocampal area of the ET-1-injected injured hippocampus was significantly increased in runners (Run) compared with non-runners (No Run). (**b**) The area of spared GCL in the dentate gyrus was reduced in the ET-1-injected hippocampus compared with the vehicle-injected contralateral hippocampus in both runners and non-runners (mean ± SEM, *n* = 8/group; paired t-tests, comparing ET-1-injected and vehicle-injected control hippocampi of the same animal, and unpaired t-tests comparing treatments; *p < 0.05; **p < 0.01).

Injection of the right hippocampus of female C57BL/6 mice with ET-1 (Fig. 1a) resulted in hippocampal shrinkage and a lesion that primarily affected the dentate gyrus, which displayed neuronal loss and localized granular cell layer thinning, whereas the CA regions were largely preserved, with no damage being observed in the vehicle-injected hippocampus (Fig. 1b–d). The total hippocampal area and the area of the spared dentate gyrus granular cell layer in the ET-1-injected right hippocampus were reduced by 18.5 ± 5.0% (paired t-test, t = 3.157, df = 7, *p* = 0.0160) and 14.3 ± 3.8% (paired t-test, t = 3.557, df = 7, *p* = 0.0093), respectively, compared with the vehicle-injected left hippocampus of control mice (Fig. 2).

To test the effects of unilateral ET-1-induced hippocampal injury on cognition, we assessed hippocampal-dependent spatial learning ability using the APA task over a 5-day testing block and, based on the number of shocks received, compared the performance of ET-1-injected animals to that of vehicle-injected controls (see Fig. 1e for experimental design; repeated measures two-way ANOVA and Bonferroni post hoc comparisons day 1 vs day 5). Prior to intrahippocampal ET-1 or PBS injection the performance of all animal groups significantly improved over the 5 days of testing as indicated by a reduction in shocks received (Fig. 1f, block APA1; F = 14.6, DFn = 4, DFd = 148, *p* < 0.0001; Bonferroni post hoc comparisons: ET-1 Injury *p* = 0.0034, Vehicle No Injury *p* = 0.0009, Naïve *p* = 0.0001) and there were no differences between the groups (F = 0.1668, DFn = 2, DFd = 37, *p* = 0.8470). There appeared to be an improvement in the maximum time animals avoided the shock-zone per session over the five days of testing (Fig. 1g, block APA1; F = 8.342 DFn = 4, DFd = 148, *p* < 0.0001). However, Bonferroni post hoc comparisons of the first and last day were not significant for any group over time). There were no differences between the groups in the maximum time they avoided the shock-zone prior to surgery (F = 0.1032, DFn = 2, DFd = 37, *p* = 0.9022).

**Figure 3:**
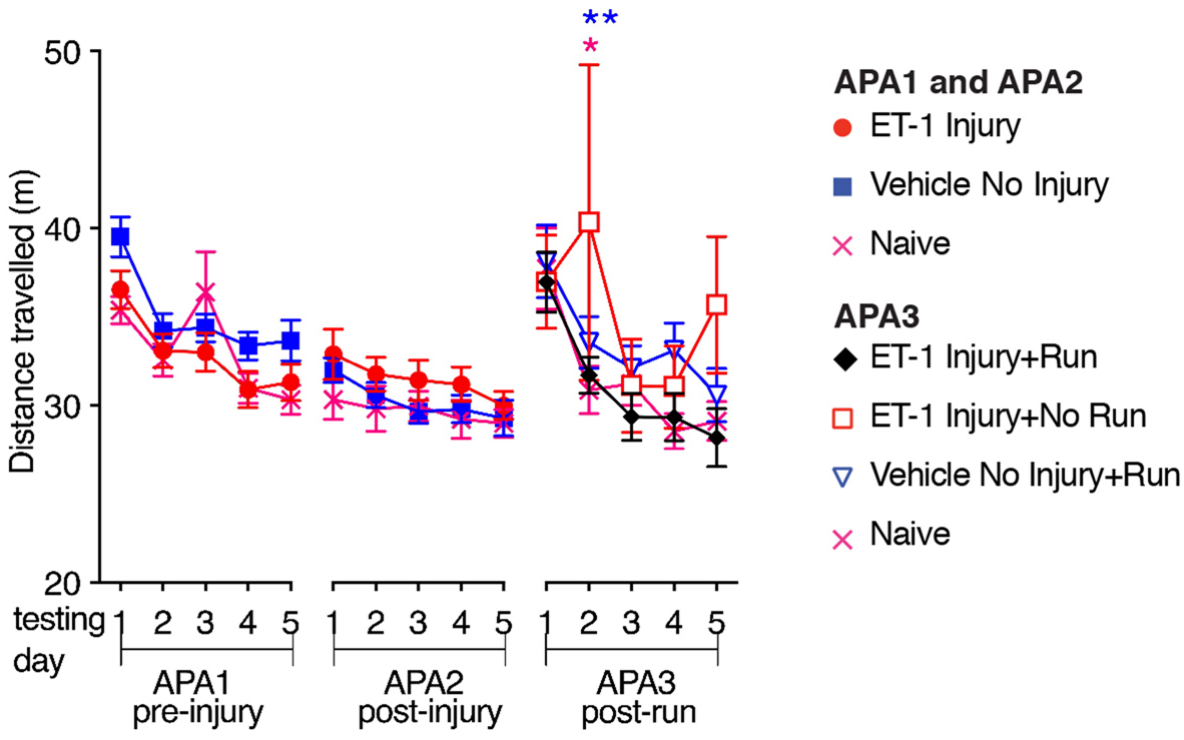
Unilateral ET-1 hippocampal injury did not affect the distance travelled in the active place avoidance task. Mice from all groups travelled similar distances in the APA task on all days of testing except for day 2 in the post-run block APA3 (mean ± SEM, *n* = 8/group; repeated measures two-way ANOVA and Bonferroni post hoc comparisons: APA 1–2 vs. ET-1 Injury; APA3 vs. ET-1 Injury + No Run; *p < 0.05; **p < 0.01).

**Figure 4:**
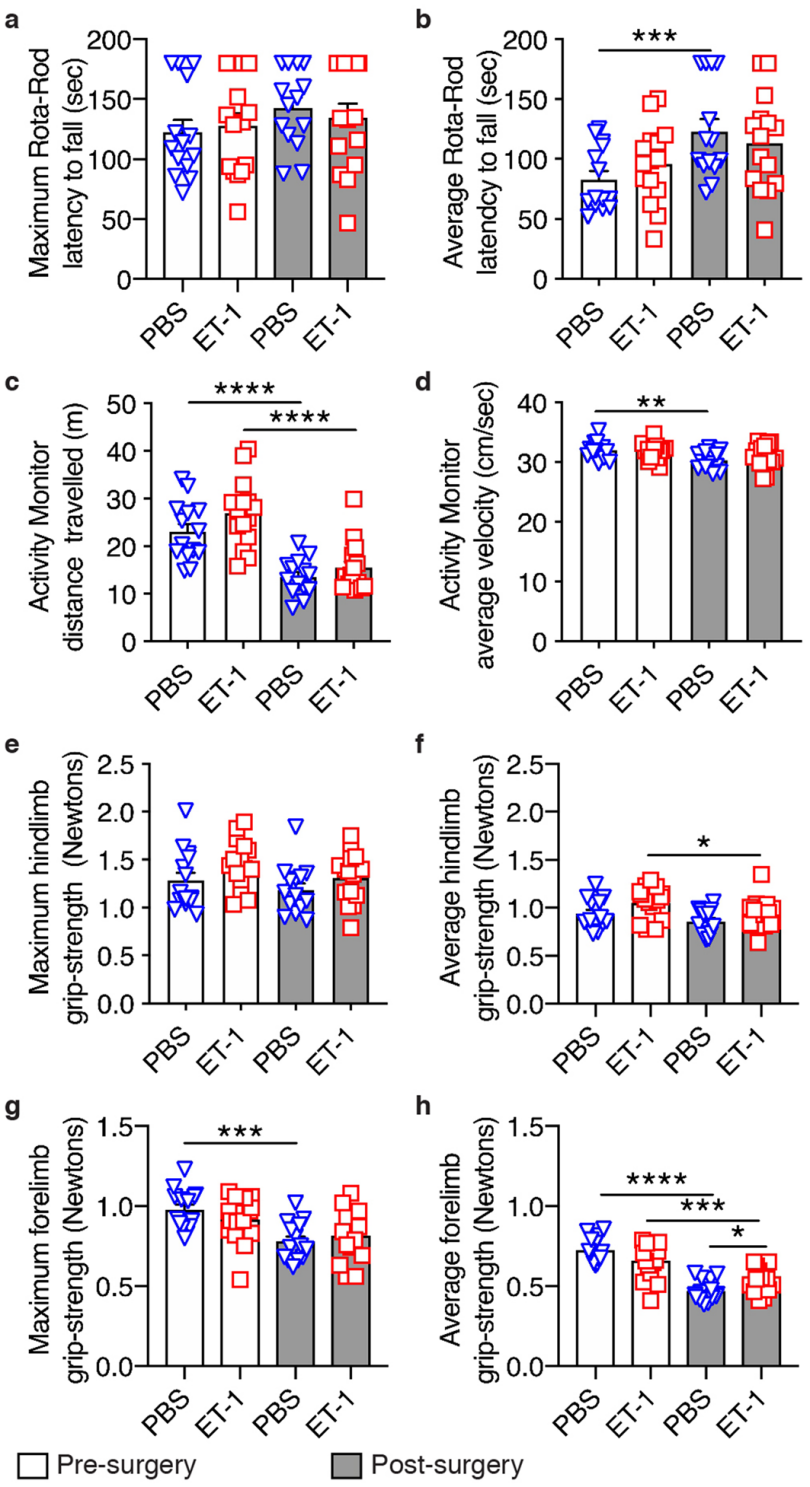
Unilateral ET-1 hippocampal injury did not impair motor function. There were no significant differences between mice receiving intrahippocampal injection of ET-1 (*n* = 14) compared with vehicle-injected controls (*n* = 15) one week after surgery in the maximum (**a**) and average (**b**) latency to fall in the Rota-Rod test (3 repeats per mouse); (**c**) the distance travelled and (**d**) average velocity in a 20 min period of activity monitoring; (**e**) the maximum and (**f**) average hindlimb grip strength (10 repeats per mouse); and (**g**) the maximum forelimb grip strength (10 repeats per mouse). However, (**h**) the average forelimb grip strength was significantly greater in the ET-1 injected stroke mice compared with the vehicle-injected mice (mean ± SEM; paired t-tests, comparing pre- and post-surgery performance, and unpaired t-tests comparing PBS- and ET-1-injection; *p < 0.05; **p < 0.01; ***p < 0.001; ****p < 0.0001).

The animals were then retested with different visual cues following unilateral ET-1 intrahippocampal injection or sham surgery (Fig. 1f, block APA2). There were no significant changes in the numbers of shocks received during the five days of testing (F = 0.4157, DFn = 4, DFd = 148, *p* = 0.7971). As vehicle-injected and naïve mice learned on the first testing day, whereas ET-1-injected animals did not learn and showed no reduction in shock numbers over the block, there were significant differences between the groups (F = 5.058, DFn = 2, DFd = 37, *p* = 0.0114). ET-1-injected mice received significantly more shocks on the final day compared with vehicle-injected or naive controls (Bonferroni post hoc comparisons: Vehicle No Injury *p* = 0.0493, Naïve *p* = 0.0084) or their pre-ET-1injection performance (paired t-test; t = 2.523, df = 15, *p* = 0.0234). There was no difference between the groups in the maximum time that animals avoided the shock-zone following unilateral ET-1 intrahippocampal injection or sham surgery (Fig. 1g, block APA2; F = 1.734, DFn = 2, DFd = 37, *p* = 0.1906). This learning deficit as evidenced by ET-1-injected animals receiving more shocks was not due to changes in motor function, as the ET-1-injected animals travelled similar distances in the APA task to mice in the vehicle-injected or naive controls (Fig. 3; F = 2.639, DFn = 2, DFd = 37, *p* = 0.0849). We confirmed that intrahippocampal injection of ET-1 did not impair motor function beyond that observed with the vehicle-injected controls in a separate group of mice using Rota-Rod, activity monitoring, and grip strength tests (Fig. 4).

### Voluntary running increases neurogenesis and rescues a unilateral ET-1 hippocampal injury-induced learning deficit

As stimulating neurogenesis has been associated with improvements in spatial learning, we therefore investigated whether the unilateral ET-1 hippocampal injury-induced learning deficit could be ameliorated by voluntary exercise. Animals were divided into cohorts with matching performance, as assessed in block APA2, and then randomly assigned to groups that either underwent voluntary running for 21 days (Run) or were maintained in standard housing (No Run). ET-1-injected animals ran similar distances as compared to the vehicle-injected animals, (0.79 ± 0.08 km and 0.97 ± 0.11 km daily, respectively; *p* = 0.2). When retested with new visual cues 2 weeks after the exercise period, the ET-1 Injury + Run animals showed remarkable recovery and performed as well as the non-injury animals that ran (Vehicle No Injury + Run; Fig. 1f, block APA3; F = 4.112, DFn = 4, DFd = 35, *p* = 0.0078). In contrast, the ET-1-injected animals that did not run (ET-1 Injury + No Run) showed further deterioration in their ability to avoid the shock-zone (Fig. 1f, block APA3; Bonferroni post hoc comparisons day 1 vs day 5, *p* = 0.0002). ET-1-injected mice that did not run received significantly more shocks on the final day compared with other groups (Bonferroni post hoc comparisons all *p* < 0.0001). The maximum time that animals avoided the shock-zone was similar between the groups (Fig. 1g, block APA3; F = 2.312, DFn = 4, DFd = 35, *p* = 0.0770) and increased over time for all groups except the ET-1-injected animals that did not run (F = 6.802, DFn = 4, DFd = 140, *p* < 0.0001). The maximum time that ET-1-injected animals that did not run (ET-1 Injury + No Run) managed to avoid the shock-zone on the final day of testing was less than that of any of the other groups (Bonferroni post hoc comparisons: ET-1 Injury + Run *p* = 0.0226, Vehicle No Injury + No Run *p* = 0.0002, Vehicle No Injury + Run *p* = 0.0253, Naïve *p* = 0.0121).

**Figure 5:**
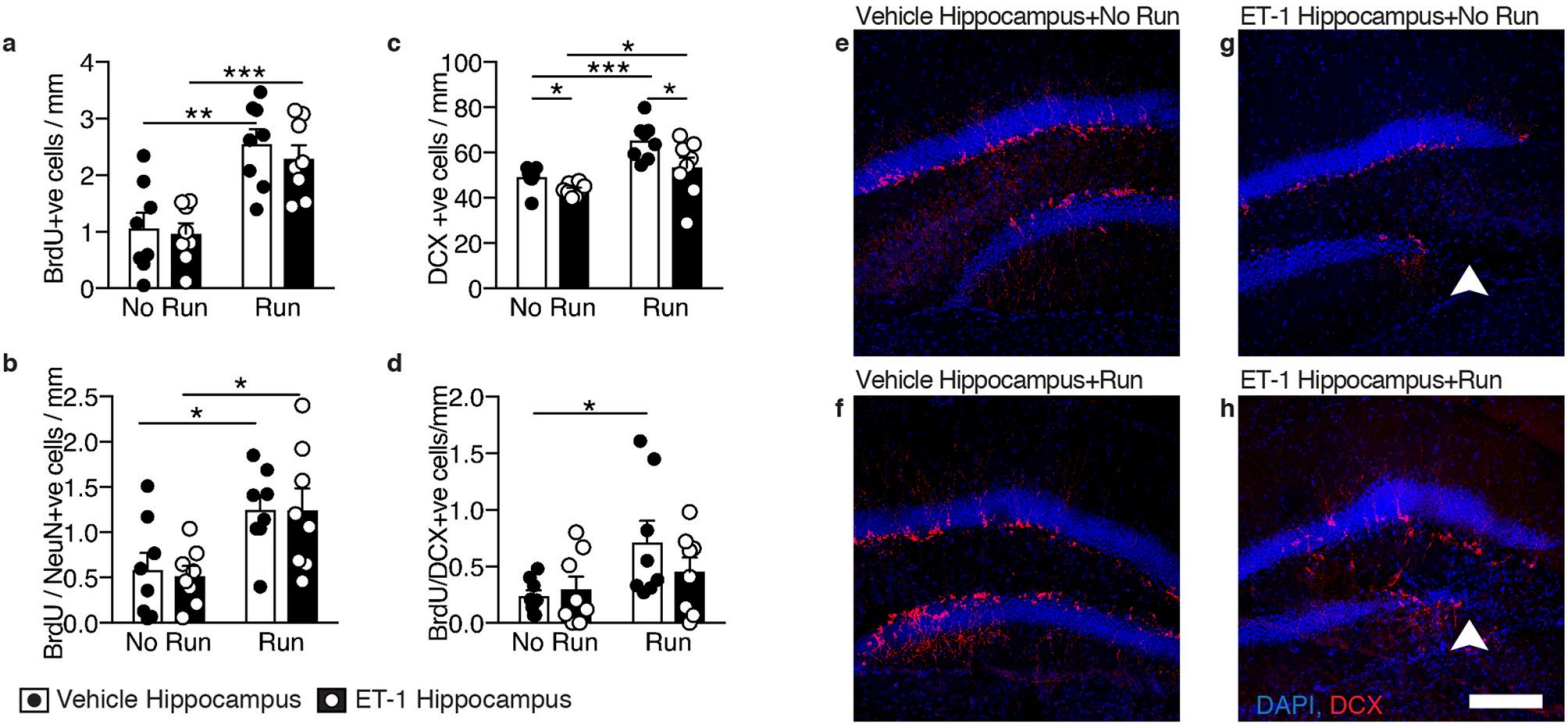
Unilateral ET-1 hippocampal injury reduced the number of DCX-positive neurons, and running increases proliferation and neurogenesis in both the vehicle-injected hippocampus and the ET-1-injected stroke hippocampus. Following unilateral ET-1 hippocampal injury, animals that ran (Run) had more BrdU-positive neurons (**a**), BrdU-positive cells co-expressing NeuN (**b**), and DCX-positive cells (**c**) in the dentate gyrus of both ET-1 and vehicle-injected hippocampi, and more BrdU-positive cells co-expressing DCX (**d**) in the vehicle-injected hippocampus than non-runners (No Run) (cell counts expressed as a density per mm length of the DG; mean ± SEM, *n* = 8/group; paired t-tests, comparing ET-1-injected and vehicle-injected control hippocampi of the same animal, and unpaired t-tests comparing treatments; *p < 0.05; **p < 0.01; ***p < 0.001). (**e–h**) There were fewer DCX-positive neurons in the dentate gyrus of the ET-1-injected hippocampus than in the contralateral hippocampus in both runners (Run) and non-runners (No Run), but more DCX-positive neurons in both hippocampi of runners compared with animals that did not run (arrowheads indicate lesion; scale bar: 100 µm).

**Figure 6:**
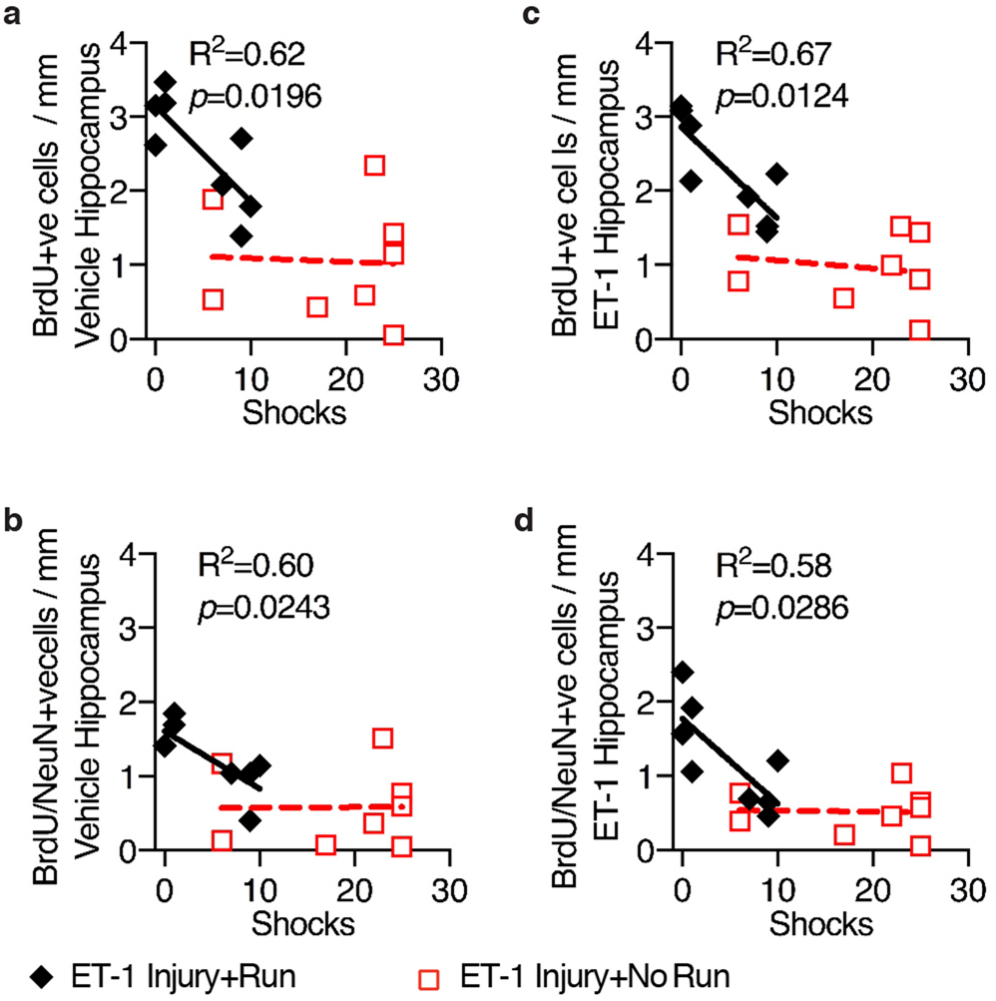
Running-induced proliferation and neurogenesis rescues the hippocampal injury-induced learning deficit. Runners with more BrdU-positive cells or BrdU-positive cells co-expressing NeuN in the dentate gyrus of vehicle-injected (**a**–**b**) and ET-1-injected (**c**–**d**) hippocampi were better able to learn and received fewer shocks on the final testing day of APA3 (mean ± SEM, *n* = 8/group; paired t-tests, comparing ET-1-injected and vehicle-injected control hippocampi of the same animal, and unpaired t-tests comparing treatments.

Given that previous studies on the effects of running after stroke have conflictingly reported either reduced^24^ or increased^25^ numbers of newborn neurons in the hippocampal dentate gyrus, we evaluated the impact of unilateral ET-1 hippocampal injury and running on hippocampal neurogenesis in our model. The animals received daily BrdU injections over the final 4 days of running, after which the number of BrdU-positive cells in the hippocampal dentate gyrus that expressed the immature neuronal marker, DCX, or the mature neuronal marker, NeuN, was assessed 3 weeks later by immunohistochemistry. The number of BrdU-positive cells (Fig. 5a), BrdU/NeuN co-expressing cells (Fig. 5b), and DCX-positive cells (Fig. 5c) in the dentate gyrus were significantly increased in both the vehicle-injected (*p* = 0.0015; 0.0181; and 0.0004 respectively; Fig. 5e as compared with Fig. 5f) and ET-1-injected (*p* = 0.0006; 0.0156; and 0.0485 respectively; Fig. 5g as compared with Fig. 5h) hemispheres in animals that ran, although there was a significant decrease in the number of DCX-positive neurons in both non-runners and runners in the ET-1-injected hippocampus compared with the vehicle-injected hippocampus (*p* = 0.0388 and 0.0348 respectively). There was also a significant increase in the number of BrdU/DCX co-expressing neurons in the vehicle-injected hippocampus in runners compared with non-runners (Fig. 5d; *p* = 0.0292). The ET-1-injected total hippocampal area was 19% larger in runners than non-runners (Fig. 2a; unpaired t-test, t = 2.424, df = 14, *p* = 0.0295). When we compared the number of BrdU-positive or BrdU/NeuN-positive cells in individual animals following running with their spatial learning performance, as measured by the numbers of shocks they received on the final testing day in the APA task, there was a significant correlation for both the vehicle- and ET-1-injected hippocampi of animals that ran (Fig. 6).

### Increased neurogenesis is required for the recovery of a unilateral ET-1 hippocampal injury-induced learning deficit

**Figure 7:**
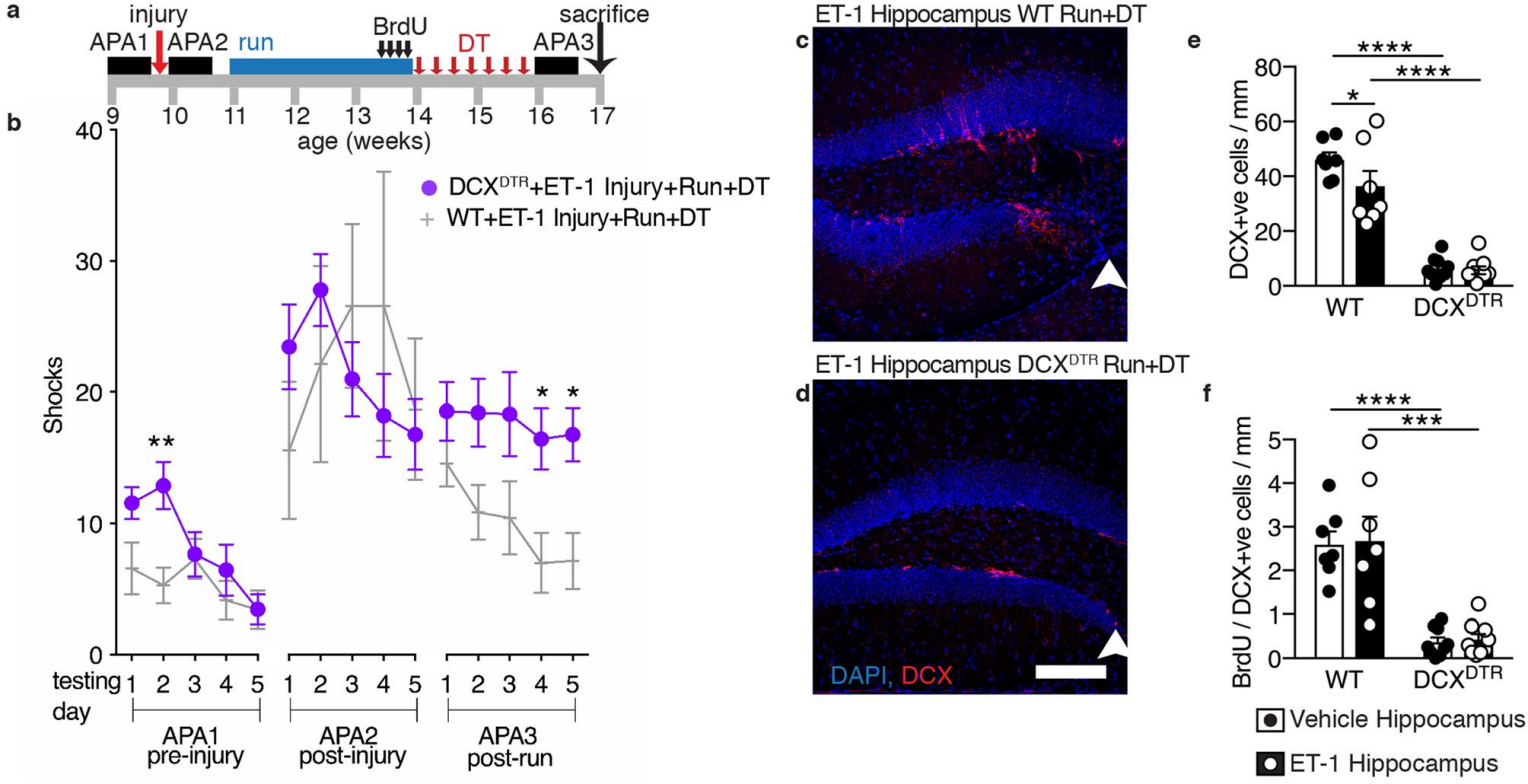
Systemic ablation of running-induced neurogenesis abrogates recovery from the unilateral ET-1 hippocampal injury-induced learning deficit. (**a**) Experimental design. (**b**) Following ET-1-induced hippocampal injury, running, and 2 weeks of diphtheria toxin (DT) intraperitoneal injections, DCX^DTR^ mice failed to learn the shock-zone location and received more shocks than wild-type (WT) mice. (**c, d)** Photomicrographs contrasting the dentate gyrus of ET-1-injected hippocampus of WT and DCX^DTR^ mice that received DT injections following running (arrowheads indicate lesion; scale bar: 100 µm). (**e, f**) DT treatment of DCX^DTR^ animals reduced numbers of DCX-positive and BrdU/DCX-positive cells compared with WT controls (cell counts expressed as a density per mm length of the DG; mean ± SEM; *n* = 7 WT and *n* = 9 DCX^DTR^ mice; **b** repeated measures two-way ANOVA and Bonferroni post hoc comparisons made over APA 1–3; **e, f** paired t-tests, comparing ET-1-injected and vehicle-injected control hippocampi of the same animal, and unpaired t-tests comparing genotype; *p < 0.05; **p < 0.01; ***p < 0.001; ****p < 0.0001).

To determine whether the improved learning was dependent on this exercise-induced neurogenesis, we used a transgenic knock-in DCX^DTR^ mouse strain we developed to specifically delete newborn neurons. DCX^DTR^ mice express the human diphtheria toxin receptor (DTR) on the surface of DCX-positive neurons, which can be selectively ablated by treatment with diphtheria toxin (DT), whereas the resident neural precursor cells remain intact^12^. Following unilateral ET-1 intrahippocampal injection, both DCX^DTR^ mice and wild-type age-matched controls on the same genetic background received intraperitoneal DT injections every second day during a 14 day post-running period (see Fig. 7a for experimental design). As immature neurons remain DCX-positive for 2–3 weeks^26^, injection of DT in the immediate post-running period ablated immature neurons produced during running and completely abolished the exercise-associated improvement (Fig. 7b, testing block APA3; F = 9.346, DFn = 1, DFd = 14, *p* = 0.0085). Immunohistochemical analysis confirmed that DCX^DTR^ animals had significantly fewer DCX-positive cells (Fig. 7c–e), and BrdU-positive cells that co-expressed DCX (Fig. 7f) in the dentate gyrus of both vehicle- and ET-1-injected hippocampi compared with wild-type animals.

### Unilateral neurogenesis is sufficient for the recovery of a unilateral ET-1 hippocampal injury-induced learning deficit

**Figure 8:**
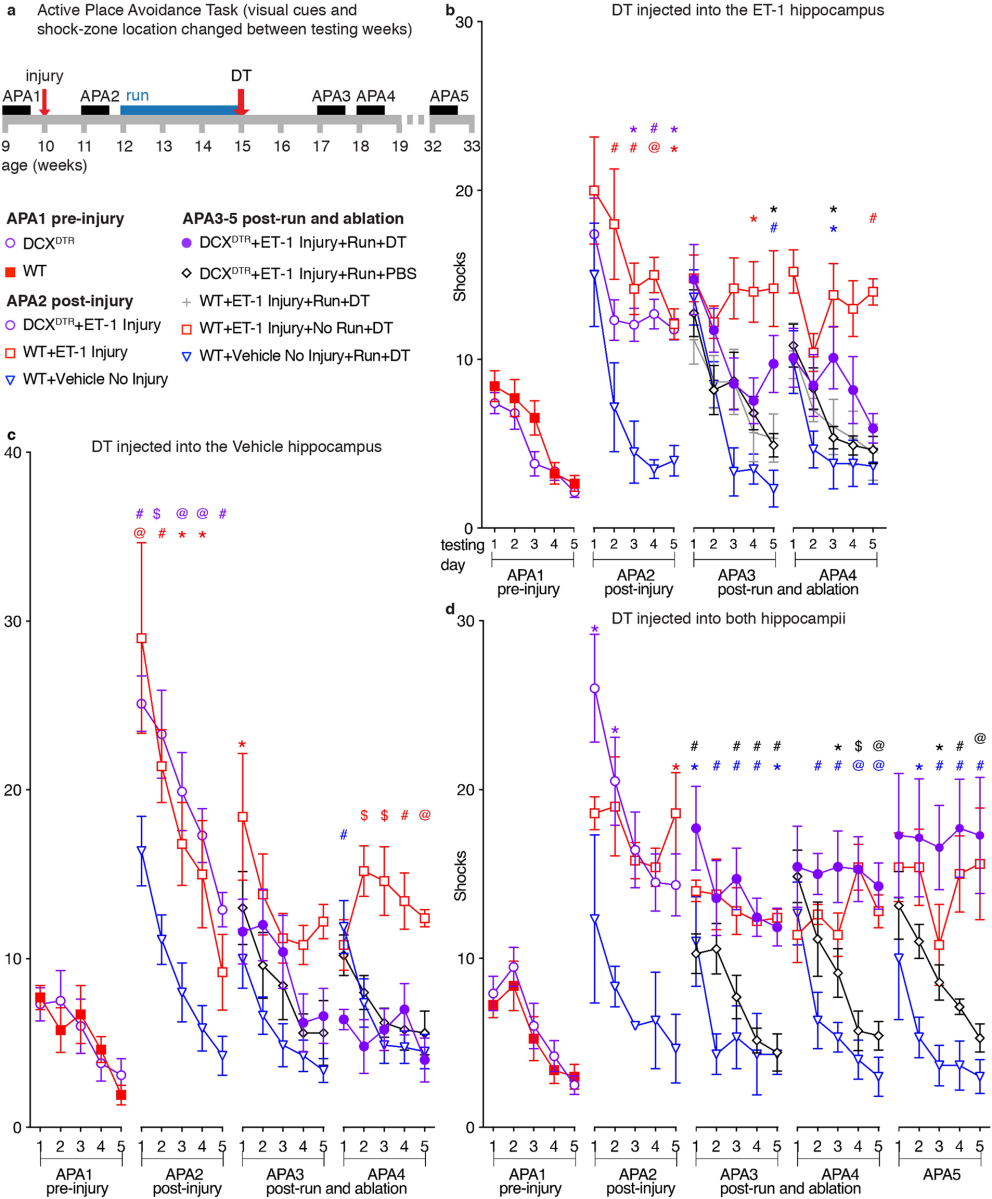
Bilateral ablation of running-induced neurogenesis by intrahippocampal injection of DT prevents recovery, whereas unilateral ablation from either hippocampus only delays recovery from a unilateral ET-1 hippocampal injury-induced learning deficit. (**a**) Experimental design. DCX^DTR^ mice injected unilaterally with DT in either the ET-1- (**b**; *n* = 11) or vehicle- (**c**; *n* = 5) injected hippocampus were unable to completely learn during APA3 and were not significantly different from WT Injury No Run mice (*n* = 5 for each condition). However, these same animals were able to learn by APA4. (**d**) Following stroke and running, DCX^DTR^ animals that received bilateral intrahippocampal DT injections (*n* = 7) were also unable to learn the shock-zone location during APA3, unlike DCX^DTR^ animals that received bilateral intrahippocampal PBS (*n* = 7) or No Injury controls (*n* = 3) and received similar numbers of shocks to WT Injury No Run mice (*n* = 5). In contrast to unilaterally injected mice, these mice never recovered, despite repeated testing (APA4–5), and were still unable to learn the shock-zone location 22 weeks after stroke (mean ± SEM, repeated measures two-way ANOVA and Bonferroni post hoc comparisons: APA 1 vs. DCX^DTR^; APA2 vs. WT + Vehicle No Injury; APA3–5 vs. DCX^DTR^ + ET-1 Injury + Run + DT; *p < 0.05; ^#^*p* < 0.01; ^@^*p* < 0.001; ^$^*p* < 0.0001; colors show the comparison group).

As running following unilateral ET-1 hippocampal injury was associated with an increase in hippocampal neurogenesis in both hemispheres, we next determined if the recovery of learning was due to increased neurogenesis in the ET-1-injected hippocampus or the result of compensation from increased neurogenesis in the vehicle-injected hemisphere. To investigate this, we developed a model whereby the number of DCX-positive neurons was reduced specifically on one side by the unilateral hippocampal injection of DT into the hilar region. This resulted in a reduction in the number of DCX-positive cells in the dentate gyrus of the injected hippocampus to 27% of that in the dentate gyrus of the non-DT-injected hippocampus 4 days after injection. We investigated the impact of unilateral ablation of DCX-positive neurons on the final day of running (see Fig. 8a for experimental design), in either the ET-1-injected (Fig. 8b) or the contralateral vehicle-injected hippocampus (Fig. 8c). DCX^DTR^ animals that received intrahippocampal injection of DT on the final day of running could not learn the shock-zone location when testing commenced 2 weeks after unilateral ET-1 hippocampal injury (APA3) and there was no significant difference in the number of shocks they received compared with wild-type ET-1 Injury + No Run mice. However, the following week (APA4), the same animals received significantly fewer shocks than wild-type ET-1 Injury + No Run mice and performed no differently from DCX^DTR^ animals that received unilateral PBS following ET-1 Injury and running, or to PBS-injected non injured wild-type controls. These results indicate that ablating the DCX-positive cells unilaterally in either the lesioned or non-lesioned hippocampus merely delayed the recovery of learning ability by one week.

We have previously reported a reduction in the number of DCX-positive cells observed in differentiated neurospheres generated from cells isolated from the subventricular zone^12^ and the systemic administration of DT would ablate DCX-positive cells in brain regions other than the hippocampus. To ensure that this delay in learning resulted from unilateral sparing of newborn immature neurons (compared with the total ablation and inability to learn observed using systemic injection of DT) and was not due to the change in protocol or non-hippocampal effects, we also performed bilateral injections of DT or PBS followed by further APA testing (Fig. 8d). Following unilateral ET-1 hippocampal injury and running, vehicle-injected DCX^DTR^ mice performed similarly to non-injured controls, learning the shock-zone location and receiving significantly fewer shocks on the final day compared with the first day of testing in block APA3. In contrast, DT-injected DCX^DTR^ Run mice performed the same as No Run mice following unilateral ET-1 hippocampal injury and received significantly more shocks than either vehicle-injected DCX^DTR^ Run mice or no-injury controls on the final testing day of APA3. Furthermore, these bilaterally injected animals did not recover their learning ability even when retested up to 22 weeks after unilateral ET-1 hippocampal injury (17 weeks after DT injection; APA4,5, Fig. 8d). These results indicate that bilateral hippocampal ablation of DCX-positive cells on the final day of running completely blocked the recovery of learning observed in animals that ran following unilateral ET-1 hippocampal injury. Therefore, increased neurogenesis in either the ET-1-injected hippocampus *or* the contralateral hippocampus is sufficient to improve recovery in learning.

## Discussion

Here we have shown that a hippocampal-mediated learning deficit associated with a unilateral ET-1-induced lesion can be rescued with voluntary running. Systemic ablation of immature neurons in DCX^DTR^ mice at the end of the running period following unilateral ET-1 hippocampal injury revealed that this improved cognitive function was mediated by the activation of hippocampal precursor cells and increased neurogenesis. This observation was further confirmed by our new technique of intrahippocampal injections of DT in DCX^DTR^ mice which enabled us to localize the ablation of immature neurons to the hippocampus. We observed that there was no functional improvement when immature neurons produced during the running period were bilaterally ablated. However, functional recovery was possible following unilateral ablation of newborn neurons.

Interestingly, improved learning occurred following unilateral ablation of newborn neurons regardless of whether they were ablated from the lesioned or non-lesioned hippocampus. We are therefore unable to confirm that the running-induced increase in neurogenesis resulted in actual repair to the damaged hippocampus. Furthermore, we never observed the complete lesion of the dentate gyrus following intrahippocampal injection of ET-1. In all animals, a portion of the dentate gyrus in the lesioned hemisphere appeared to contain healthy mature neurons and proliferating cells as evaluated with immunohistochemistry. If unilateral ablation of newborn neurons produced during the running period from the ET-1-injected hemisphere had prevented recovery in APA performance, it would indicate that a repair mechanism was required. However, it seems more likely that compensation from newborn neurons integrating into spared dentate gyrus tissue in the lesioned hippocampus *or* greater numbers of newborn neurons integrating into the non-lesioned hippocampus was sufficient for the recovery in APA performance. Running has also been found to induce hippocampal and entorhinal cortical structural changes^27^. Long-term voluntary runners have increased dendritic spine density in different regions and altered dendritic arborization in the CA1 region. Although we did not examine these properties, we did observe an increase in the total hippocampal area of the ET-1-injected hemisphere of runners compared with non-runners, which may be indicative of structural changes.

Although not frequently the site of the primary stroke^28^, the reduction in hippocampal volume reported in humans with cortical or subcortical lesions has been associated with impaired cognition^2–4^. In order to investigate treatments that have the potential to improve post-stroke cognitive outcomes, accurate evaluation of hippocampal-dependent learning function is a necessity. Despite being the most commonly used model in pre-clinical stroke research, the MCAO model results in motor-function deficits that confound such an evaluation. Both the 1999 recommendations from the Stroke Therapy Academic Industry Roundtable (STAIR)^22^ and the 2009 updated recommendations^23^ recommended that, to better reflect conditions observed in human strokes, the permanent MCAO model should be utilised in initial experiments followed by transient occlusion models. These recommendations are largely in the context of thrombolysis and neuroprotection studies.

This series of experiments was designed to examine the role of hippocampal neurogenesis in cognitive recovery following hippocampal damage. Global ischemia models, such as bilateral common carotid artery occlusion, reportedly result in a bilateral loss of CA1 hippocampal neurons in C57BL/6 mice due to an abnormality in that strain’s cerebrovasculature, but produce less consistent results in other mouse strains^29–33^. Furthermore, recovery treatments require longevity to be relevant^22^. Our ET-1 injection model results in unilateral hippocampal damage, no observable motor function deficits, and long term survival. Whilst this study does not comply with the STAIR recommendation to use the permanent MCAO model, our ET-1 injection model has allowed us to examine post-injury changes in hippocampal-dependent learning and the role of neurogenesis in cognitive recovery over an extended time period. Our observations that a hippocampal-mediated learning deficit associated with a unilateral ET-1-induced lesion can be rescued with voluntary running is a preliminary study in young female mice with no co-morbidities. To meet the STAIR recommendations regarding relevance to human populations, these studies should be repeated in aged animals with comorbidities as well as in different species. Future studies in higher-order animals should also examine if there are any changes in white matter^23^.

Activatable hippocampal precursor cells represent novel therapeutic targets for improving cognitive function following hippocampal injury. Exercise, enriched environments, and socialization have been found to stimulate neurogenesis in the normal adult mammalian brain. However, investigations into the responsiveness of these precursor cells in the environment of the injured brain have been less compelling. Following global ischaemia induced by four-vessel occlusion in rats, 14 days of voluntary running treatment was found to reduce the survival of cells that had been labelled prior to running^24^, although cognitive evaluations were lacking in this study. In contrast, newborn DG cell survival was reportedly enhanced, and spatial memory impairment was reversed in mice subjected to MCAO that were allowed voluntary running for 21 or 42 days, a response that was not observed when the animals underwent forced swimming^25^. Voluntary exercise has also been shown to rescue cognitive deficits in rats subjected to a mild fluid percussion injury^17^.

The mechanism whereby running stimulates increased levels of neurogenesis is yet to be comprehensively determined, although changes in the levels of several factors have been identified. Running has been reported to lead to increased levels of growth hormone^34^, brain-derived neurotrophic factor (BDNF)^35^, microRNA-124 and tyrosine kinase B^36^, and has also been associated with reduced levels of RE1-silencing transcription factor^36^. The recovery from cognitive deficits suffered by adult rats subjected to a mild fluid percussion injury that then underwent voluntary exercise was also been shown to be mediated by BDNF^17^. It has been reported that serotonin has a direct involvement in regulating hippocampal neurogenesis induced by exercise in both young and aged mice^37^. As many stroke survivors or people living with neurological disorders are unable to exercise due to ongoing mobility issues, these molecular targets may represent alternative approaches to stimulating neurogenesis to improve cognitive recovery. However, given that there is already evidence that exercise not only increases hippocampal volume but also improves spatial memory^38^ and cognition^39^ in older adults, voluntary exercise in physically able survivors represents a relatively simple and non-invasive treatment to stimulate endogenous neurogenesis, and may improve recovery from the learning deficits commonly observed in humans following stroke and in other neurodegenerative disorders.

## Methods

### Animals

Experiments were conducted in accordance with the Australian Code of Practice for the Care and Use of Animals for Scientific Purposes, with approval from The University of Queensland Animal Ethics Committee. Female C57BL/6 and DCX^DTR^ mice (aged 10–13 weeks at ET-1 intrahippocampal injection surgery) were used in these experiments. DCX^DTR^ mice^12^ have the human diphtheria toxin receptor (DTR) inserted under the control of the doublecortin (DCX) promoter.

### Experimental timeline

Schematics of the relevant experimental design and timelines are displayed in Figs. 1, 7 and 8. In the first experiment, mice underwent the active place avoidance (APA) task, a test of hippocampal-mediated learning, before (APA1: habituation day − 7, testing days − 6 to − 2) and after (APA2: days 2 to 6) ET-1 injections or sham surgery (day 0). They were then housed in standard cages with or without a running wheel for 21 days (days 8 to 28). To evaluate proliferative and neurogenic rates *in viv*o, the mice received intraperitoneal injections of bromodeoxyuridine (BrdU; Sigma-Aldrich; 100 mg/kg) on the final 4 days of running (days 25 to 28). They then underwent further APA retesting (APA3: days 43 to 47), were sacrificed (day 49), and immunohistochemistry and cell counts in brain sections were performed. The timeline for the intraperitoneal diphtheria toxin (DT) injection was identical, except that animals received intraperitoneal injections of DT on alternate days starting on day 31 post-ET-1-injection. For the intrahippocampal DT experiments, the timeline was similar to that of the first experiment except that running occurred from days 9 to 29, BrdU was injected on days 26 to 29, and animals received a single intrahippocampal injection of DT (10 pg/0.5 µl phosphate buffered saline: PBS) on the final day of running (day 29).

### ET-1-induced hippocampal injury and intrahippocampal DT surgery

The posterior cerebral artery (PCA) is the main supplier of blood to the hippocampus via the transverse hippocampal arteries that pass through the hippocampal fissure^40^, and we know of no model that successfully clamps the PCA or hippocampal branches. We therefore injected the vasoconstrictor endothelin-1 (ET-1)^41^ directly into the hippocampal fissure. Female C57BL/6 and DCX^DTR^ mice (aged 10–13 weeks at intrahippocampal injection) were anesthetized by intraperitoneal injection of a mixture of ketamine hydrochloride (100 mg/kg; Parnell Laboratories) and the muscle relaxant xylazine (10 mg/kg; Troy Laboratories), before being placed in a stereotaxic apparatus (David Kopf Instruments). Two small holes were drilled in the skull over the hippocampus. One microliter of ET-1 (Merck Pty Ltd) containing Fluororuby (at a final dose of 333 pmol ET-1) was injected into the right hippocampus and 1 μl of PBS was injected into the left hippocampus. Both injections were made directly into the hippocampal fissure, at the following coordinates relative to Bregma (anteroposterior: − 1.8 mm; mediolateral: ± 1.0 mm; dorsoventral: − 1.7 mm). These injections occurred over 5 min via a pulled borosilicate glass micropipette (1/0.58 mm outer/inner diameter; World Precision Instruments) attached to a 5 µl Hamilton syringe. To reduce backflow, the pipette was left in place for a further 10 min before being withdrawn slowly over 2 min. Sham surgery animals were bilaterally injected with PBS. Intrahippocampal injection of DT was made into the hilus at slightly different coordinates relative to Bregma (anteroposterior: − 2.0 mm; mediolateral: ± 1.5 mm; dorsoventral: − 2.0 mm). Animals were placed in a humidified incubator at 33ºC for two days following ET-1-intrahippocampal injection or until they were alert following DT intrahippocampal injection, after which they were housed together (three to four mice per cage) at room temperature. At the time of surgery and for the following 2 days mice were given intraperitoneal injections of the analgesic torbugesic butorphanol tartrate (Fort Dodge, Provet; 2 mg/kg), as well as the antibiotic enrofloxacin (Bayer Australia; 5 mg/kg) in saline.

### Active place-avoidance behavioral testing of hippocampal-mediated learning

The APA task was conducted as previously described^12^. The equipment (Bio-Signal Group) consisted of an elevated motorized wire platform with a clear Perspex cylinder (height: 32 cm; diameter: 77 cm). A 60º segment of the cylinder was defined by a computer program as the shock-zone, and this shock-zone remained stationary relative to the visual cues placed on the walls. The animal was placed in the cylinder opposite the shock-zone and the platform was rotated at 1 revolution per minute (rpm). The position of the animal was determined by Track Analysis software (Biosignal) and an overhead camera. If the animal remained motionless, the rotating platform moved the animal into the shock-zone. Although the platform was rotating, the relationship between the shock-zone and the visual cues was kept constant by the software, such that the mouse had to actively avoid the shock-zone by learning its location using the visual cues on the walls. If an animal entered and remained in the shock-zone for 0.5 s it received a brief foot shock through the wire platform (500 ms, 0.5 mA, intershock interval 1.5 s) until it exited this zone. The initial testing block consisted of 1 day of habituation (5 min in a rotating arena without shocks) followed by 5 days of testing (10 min/day). The habituation session was not repeated for subsequent testing blocks. Room luminosity was set at approximately 100 lx. Animal movements and shock delivery were recorded and analyzed using Track Analysis software (Biosignal). The visual cues placed on the walls were changed for each 5 day block of testing. For the first experiment, the shock-zone position relative to the room coordinates remained the same for each 5 day block of testing; however, in subsequent experiments the shock-zone location was varied between each testing block. Excrement was removed and the equipment cleaned with 70% ethanol between trials. The APA test has previously been used in rats following temporary hippocampal inactivation with tetrodotoxin, which reportedly prevented spatial memory formation^42^. We have shown that the APA task is highly reproducible and dependent on neurogenic activity in the dentate gyrus^12^, whereas the Morris Water Maze is inconsistent in detecting post-stroke hippocampal deficits^43,44^.

### Motor function tests

Motor function tests were performed prior to surgery and one week after intrahippocampal injection of ET-1 or PBS.

### Rota-rod performance

Motor function was evaluated with an accelerating Rota-Rod apparatus (Ugo Basile) by measuring the latency to the first fall when animals were placed on an accelerating rotating rod. Animals performed three trials of 3 min each, at a minimum 3 rpm to a maximum 30 rpm, with an acceleration period of 1.5 min. The latency to the first fall was averaged over the three trials.

### Activity monitoring

Open field locomotor activity was evaluated via automated Activity Monitor analysis (Med Associates). Briefly, animals were placed in the center of an arena (27 ×  27 ×  20.3 cm) and parameters including distance travelled and average velocity were recorded over a 20 min trial.

### Grip strength

Forelimb and hindlimb grip strength were evaluated using a Digital Force Gauge (Ugo Basile; 10 trials per mouse). Grip strength was averaged over the ten trials.

### Voluntary running

To determine if voluntary running stimulated neurogenesis and improved functional recovery after unilateral ET-1 hippocampal injury, mice were housed three to four per cage and allowed 21 days of free access to an “angled-disc-type” wireless rodent running wheel, with collective distances run in each cage tracked by Wireless Running Wheel Manager Data Acquisition Software (Able Scientific). After 21 days running, the running disc was removed from the home cage.

### Ablation of DCX-positive cells

To ablate DCX-positive cells, we used transgenic DCX^DTR^ mice^12^ with the human DTR inserted under the control of the DCX promoter. Newborn DCX-positive neurons were selectively ablated with DT. For systemic ablation, female DCX^DTR^ mice and age-matched wild-type controls on the same C57BL/6 background were given intraperitoneal injections of 10 ng DT (Sigma) per gram of bodyweight every second day for 14 days (total of seven injections), 31–43 days following unilateral ET-1 hippocampal injury. This was followed by behavioral testing on days 44–48 and sacrifice on day 48.

For ablation of DCX-positive neurons by intrahippocampal DT injection following unilateral ET-1 hippocampal injury and running, female DCX^DTR^ and wild-type age-matched controls received intrahippocampal injections of DT (10 pg/0.05 µl PBS) or PBS the day the running wheels were removed. This was followed by behavioral testing at 49–53 and 56–60 days after unilateral ET-1 hippocampal injury, as well as at 154–158 days after unilateral ET-1 hippocampal injury, in the case of the bilaterally injected groups.

### Immunohistochemistry, lesion evaluation, and cell counts

Mice were overdosed with sodium pentobarbitone and transcardially perfused with mouse tonicity PBS followed by 4% paraformaldehyde (PFA) (Sigma-Aldrich) in PBS. Whole brains were removed and post-fixed overnight at 4ºC in 4% PFA. Brains were cryoprotected in 30% sucrose at 4ºC for 2 days and serial 40 μm coronal sections were cut using an SM2000R microtome (Leica). Sections were stored in PBS with 0.01% sodium azide (Sigma-Aldrich). One series of every sixth section encompassing the entire hippocampus was subjected to immunohistochemistry for BrdU and the immature neuronal marker DCX. The adjacent series underwent immunohistochemistry for BrdU and NeuN, a mature neuronal marker. Free-floating sections were incubated in 1 M HCl at 45ºC for 40 min, followed by PBS washes (3 × 5 min). Sections were incubated at room temperature for several hours with a blocking solution of 3% (v/v) normal goat serum (Sigma-Aldrich) and 0.1% (v/v) Triton X-100 (Sigma-Aldrich) in PBS. They were then incubated overnight at 4ºC in fresh blocking solution with the following primary antibodies in different combinations: rat anti-BrdU (1:500; Accurate Chem and Sci Co), rabbit anti-DCX (1:500; Abcam), or mouse anti-NeuN (1:100; Millipore). Sections were washed in PBS (3 × 10 min) and incubated for 2 h at room temperature in PBS with DAPI (1:1,000) and secondary Alexa-Fluor (anti-mouse-A647, anti-rabbit-A647, anti-rat-A488) antibodies (all 1:1,000 and raised in goat; Invitrogen). Sections were washed in PBS (3 × 5 min) and mounted with ProLong Gold (Invitrogen).

Seven 40 µm sections in series (240 µm apart) were evaluated per animal. Lesion evaluation and cell counts were conducted by an investigator blinded to treatment/genotype. Sections were visualized using a Zeiss Imager.Z2 connected to a motorized MAC 6000 System stage (MBF Biosciences). To evaluate the lesion size, DAPI and NeuN staining were used to delineate damaged regions, defined as regions where the granule cell layer (GCL) was obviously thinned from cell loss or consisting of > 90% pyknotic cells. The inner length and area of the GCL of the dorsal and ventral dentate gyrus blades and the area of the entire hippocampus were measured at 10X magnification using StereoInvestigator 10.53 (MBF Bioscience-MicroBrightField Inc.). Cell counts were performed at 40X magnification focusing throughout the entire Z-axis and for 100% of the dentate gyrus in each of the seven sections. Data were accessed with Neurolucida Explorer 10.52. DCX-positive, BrdU-positive, DCX/BrdU double-positive, and BrdU/NeuN double-positive cells were counted in the upper and lower blades of both the vehicle-injected control hippocampus and the ET-1-injected hippocampus and expressed as a density per mm length of the DG. Representative images were acquired using an Axio-Imager (Zeiss).

### Experimental design and statistical analysis

Data are expressed as mean ± SEM. Data were analyzed using GraphPad Prism version 8.4.1 for Mac OS X and all statistical tests were two-tailed. APA behavioral data (including distance travelled) were analyzed using a repeated measures two-way ANOVA and Bonferroni post hoc comparisons comparing the relevant experimental condition to other conditions. The experimental conditions used for multiple comparisons were as follows:

**Table:** 

Figures 1 and 3	APA1, 2: ET-1 Injury
	APA3: ET-1 Injury + No Run
Figure 7	APA1–3: DCX^DTR^ + ET-1 Injury + Run + DT
Figure 8	APA1: DCX^DTR^
	APA2: WT + Vehicle No Injury
	APA3–5: DCX^DTR^ + ET-1 Injury + Run + DT

In addition, behavioral data were also analyzed using a repeated measures two-way ANOVA and Bonferroni post hoc comparisons comparing the numbers of shocks received by experimental groups on each day of testing with those received on the first day of testing for each APA block. Cell counts were evaluated with paired t-tests, comparing the ET-1-injected and the vehicle-injected control hippocampus of the same animal, or unpaired t-tests to compare treatments and genotypes. Motor function tests were analyzed using paired t-tests, comparing pre- and post-surgery performance, and unpaired t-tests comparing PBS- and ET-1-injection.

The minimum animal numbers required were determined by the appropriate power calculations (α of 0.05 and power of 0.80) from preliminary experiments. Animals were divided into experimental groups with similar APA performances prior to intervention and then these groups were randomly assigned to an experimental condition (e.g. Injury vs No Injury or Run vs No Run). Researchers were not blinded during APA behavioural experiments as data collection was automated. All cell counts were conducted by researchers blinded to the experimental condition.

## Data Availability

All data generated or analyzed during this study are included in this published article.
